# Diabetic Retinal Grading Using Attention-Based Bilinear Convolutional Neural Network and Complement Cross Entropy

**DOI:** 10.3390/e23070816

**Published:** 2021-06-26

**Authors:** Pingping Liu, Xiaokang Yang, Baixin Jin, Qiuzhan Zhou

**Affiliations:** 1 College of Computer Science and Technology, Jilin University, Changchun 130012, China; jinbx18@mails.jlu.edu.cn; 2 Key Laboratory of Symbolic Computation and Knowledge Engineering of Ministry of Education, Jilin University, Changchun 130012, China; 3 School of Mechanical Science and Engineering, Jilin University, Changchun 130025, China; 4 College of Software, Jilin University, Changchun 130012, China; yangxk20@mails.jlu.edu.cn; 5 College of Communication Engineering, Jilin University, Changchun 130012, China; zhouqz@jlu.edu.cn

**Keywords:** fine-grained image classification, attention mechanism, bilinear pooling model

## Abstract

Diabetic retinopathy (DR) is a common complication of diabetes mellitus (DM), and it is necessary to diagnose DR in the early stages of treatment. With the rapid development of convolutional neural networks in the field of image processing, deep learning methods have achieved great success in the field of medical image processing. Various medical lesion detection systems have been proposed to detect fundus lesions. At present, in the image classification process of diabetic retinopathy, the fine-grained properties of the diseased image are ignored and most of the retinopathy image data sets have serious uneven distribution problems, which limits the ability of the network to predict the classification of lesions to a large extent. We propose a new non-homologous bilinear pooling convolutional neural network model and combine it with the attention mechanism to further improve the network’s ability to extract specific features of the image. The experimental results show that, compared with the most popular fundus image classification models, the network model we proposed can greatly improve the prediction accuracy of the network while maintaining computational efficiency.

## 1. Introduction

**Figure 1:**
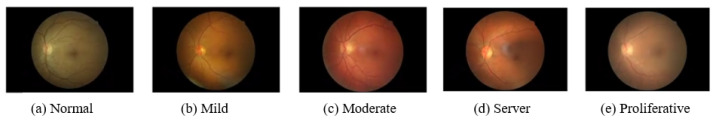
Five types of lesion grades in the data set.

Diabetic retinal fundus disease is a common ophthalmological disease in diabetic patients. Images of fundus lesions can be divided into five levels according to the degree of fundus lesions: normal, mild, moderate, severe, and hyperplasia [1]. Figure 1 shows the five levels of diabetic fundus lesions. Optical coherence tomography (OCT) is a noninvasive medical imaging technology developed in recent years. The image generated by OCT has a high resolution, and doctors can classify it according to the characteristics of the lesion in the image. The fundus images of patients with diabetic retinopathy need to be collected by professional equipment and then clinically diagnosed by the doctor’s observation. It usually takes several days to wait for the results, which prolongs the time for doctor–patient communication and even delays the condition. Due to the variety of lesions and the difficulty in quantifying the classification conditions, it is often inaccurate and difficult to manually determine the classification of lesions. At present, most DR classification tasks are still completed by doctors, which undoubtedly caused a lot of waste of manpower and material resources.

In order to solve the above problems, it is necessary to create a computer-aided diagnosis system to use a unified measurement standard to judge the level of lesions. In recent years, with the development of artificial intelligence and computer technology, computer technology has been integrated with various disciplines. Computer technology has promoted the development of many interdisciplinary subjects. Among them, medical image processing is one of the interdisciplinary subjects between medicine and computer technology [2]. The use of computer science image processing technology, computer vision and other methods to carry out a standard analysis of medical images helps to unify the results of medical diagnosis. Many medical image classification methods based on deep learning have been widely used in the field of diabetic retinopathy retinal image classification. Kaggle also initiated several competitions on the automatic detection of diabetic retinopathy. Diabetic retinal fundus image classification is different from general image classification. The image features in the diabetic fundus disease data set have a high degree of similarity (only different in some lesions such as bleeding points or aneurysms). The same diabetic retinal fundus lesions may cause different grades of lesions, which lead to similarity of the images of different grades of lesions. Therefore, the image classification of diabetic retinal fundus lesions can be regarded as a fine-grained classification problem. Traditional image classification focuses on the distinction between categories. It can achieve good results on data sets with large differences between categories (such as cats and dogs, snakes and frogs). However, fine-grained image classification further divides images of the same category, which requires the network model to effectively extract the distinctiveness of the data set. Traditional network models cannot effectively classify images with fine-grained attributes.

**Figure 2:**
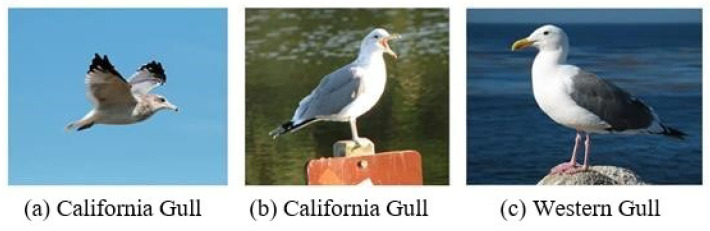
Fine-grained image classification of gulls.

Figure 2 shows two kinds of birds in the data set CUB-200 [3]. They are very similar in appearance. Among them, (a) and (b) belong to the California gull while (c) belongs to the Western gull. Although (a) and (b) belong to the same kind of bird, (b) is quite different from (a) in appearance. In fact, (b) is more similar to another type of bird (c). The greater difference within the same category than between different categories is a characteristic and a challenge of fine-grained classification. The unique characteristics of fine-grained classification make it difficult for traditional convolutional neural network models to effectively identify fine-grained classified images.

**Figure 3:**
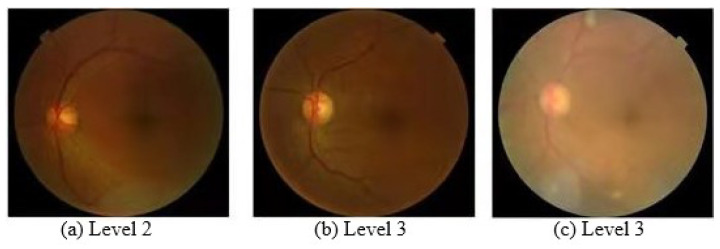
Fine-grained image classification of diabetic retinal fundus disease.

In the process of analyzing the data set of diabetic retinal diseases, we found that the classification of diabetic retinal diseases can be solved from the perspective of fine-grained image classification. As shown in Figure 3, the image (b) belongs to the level 3 fundus disease, but it is not similar to the image of the same level (c). On the contrary, (b) and (a) are more similar even though they belong to different levels. The above characteristics of the data set images make it difficult to classify retinopathy images and it is easy to confuse the classification of adjacent class images. The traditional network model cannot accurately distinguish this kind of data with fine-grained attributes. In view of the similarity between the classification of diabetic retinal fundus disease and the fine-grained classification, we used a compact bilinear pooling network model combined with the attention mechanism to obtain more distinguishable image features. The compact bilinear pooling model is a commonly used network model in the field of fine-grained classification. It uses two feature extractors to extract image features. The image features obtained by the two feature extractors are merged through a bilinear pooling operation, which enables the bilinear network model to extract more image features. The attention mechanism is mainly used to locate the key areas in the data set image, so that the network can pay more attention to useful information and weaken useless information. By combining the attention mechanism with the bilinear pooling network model, the classification accuracy of the network model can be further improved. Experiments show that the combination of bilinear pooling operation and attention mechanism can effectively solve the problem that traditional networks cannot effectively classify diabetic retinopathy images.

Similar to real medical scenarios, the proportion of severely ill patients in the diabetic retinal fundus disease image data set accounts for a very small portion of the total number of people examined, which causes the acquired data set to have a serious unbalanced distribution problem. The unbalanced data distribution increases the difficulty of training the network model, making the trained network model not robust enough. When using these unbalanced data sets to train the network, the category with a larger number of samples dominates the training process, which causes the network to have a huge difference in the performance of the categories with a different number of samples. The network performs well on categories with a large sample size but performs poorly on categories with a small sample size. Focal loss [4] is mostly used in the grading methods of diabetic retinal fundus lesions in the past to alleviate the impact of unbalanced sample distribution. Focal loss used the modulation factor in the loss to focus the model on the training of difficult samples, and focal loss also affects the imbalance of the data set by adjusting the weight. However, it cannot solve the problem of simple and difficult samples. For this reason, this paper proposes a new loss function based on complementary entropy and cross-entropy. Since the method is driven by training samples of non-labeled categories, it can use incorrect class information to train a robust classification model with unbalanced class distribution. Therefore, this method can alleviate the impact of uneven data distribution on the predictive ability of the network model without increasing the number of samples in a few categories. This strategy provides better learning opportunities for a few types of samples and makes the network model pay more attention to diseased samples to improve the recognition ability of the network model.

## 2. Related Work

The aim of research on the classification of diabetic retinal fundus disease is to classify patients according to the degree of disease from an image of the fundus of the patient. This research can help doctors quickly confirm the patient’s lesion grade and the location of the lesion area so that doctors can perform the next step of treatment for the patient according to the patient’s lesion type and severity.

The current research on the classification of diabetic retinal fundus lesions can be divided into two categories: research methods based on machine learning and methods based on deep learning. The method based on machine learning require researchers with a medical background to manually extract the image features in the diabetic retinal fundus disease data set. The classification model only needs to classify the image based on the extracted features. As early as 1996, Nguyen et al. [5] proposed the use of a multi-layer feedforward network to classify the degree of diabetic retinopathy. Zhang et al. [6] used fuzzy C-means clustering algorithm and support vector machine to classify lesion features. Subsequently, they proposed another method of combining dimensionality reduction with support vector machines to learn the characteristics of bleeding points and to classify the disease according to the characteristics of bleeding points [7,8]. Barriga et al. [9] analyzed the retinal area near the center of the image and used amplitude modulation–frequency modulation for feature extraction and used least squares and support vector machines for classification. Xu et al. [10] proposed a feature extraction method based on stationary wavelet transform and gray level co-occurrence matrix to identify exudates and to achieve diabetic retinopathy classification through support vector machines. Sinthanayothin et al. [11] proposed a KNN algorithm based on morphological features to classify diabetic retinopathy. Acharya et al. [12] used HOS technology to extract parameters from the original image and used a support vector machine classifier to classify five fundus images.

The method based on machine learning requires experts to perform manual feature design, and feature extraction also requires a large number of feature annotations, which consume a lot of effort, material resources, financial resources, and time. The method based on deep learning does not require too much manual processing of the feature extraction algorithm. The deep learning method can extract deeper image features and the relationship between these features through methods such as convolutional neural networks. Therefore, in the grading model of diabetic retinal fundus disease, deep learning algorithm has gradually become the method adopted by most models. Pratt et al. [13] introduced a CNN-based method to identify microaneurysms, exudates, and bleeding characteristics to achieve automatic data expansion and lesion grading. Kori et al. [14] used ResNet and DenseNet to achieve diabetic retinopathy classification. Torrey et al. [15] developed a more interpretable CNN model to detect lesions in retinal fundus images. Wang et al. [16] added a regression activation feature map (RAM) after global average pooling. This model can locate the distinguishable area of the retinal image and can automate the classification of diabetic retinal fundus lesions. Yang et al. [16] introduced an unbalanced weighted mapping method to achieve lesion grading, which can point out the location of the lesion. Bravo et al. [17] used a network based on VGG-16 and Inception-4 to construct a hierarchical model of diabetic retinopathy. Although these deep learning methods have achieved significant results, the fine-grained properties of the fundus images of diabetic retinopathy have not been discovered and the specificity of the features extracted by the model is insufficient, resulting in insufficient classification performance.

As the diabetic retinal fundus disease image variable data set has the characteristics of fine-grained image classification, the traditional convolutional neural network model cannot effectively extract the distinguished specific features in the image.The deep convolutional network-based fine-grained image classification is roughly divided into the following four directions [18]: detection methods based on target blocks, fine-tuning methods based on conventional image classification networks, methods based on visual attention mechanisms, and methods based on fine-grained feature learning. The fine-tuning method based on conventional image classification network uses common CNN to perform fine-grained image classification [19,20,21,22,23]. These classification networks can achieve better results in conventional image classification tasks, but they do not perform well in fine-grained image classification tasks. The detection method based on the target block detects the location of the target and the key area in the image. Then, the target image and the key area are sent into the deep network for classification at the same time. However, this method requires a lot of annotation information and key feature point information in training. It is very difficult to obtain these annotation information in medical images. The method based on the visual attention mechanism is different from the abovementioned method. This method does not require a large amount of annotation information. The attention mechanism can obtain regions of interest through self-learning methods [18], avoiding additional manual labeling information, which makes it widely used in the field of fine-grained image classification. The method based on fine-grained feature learning extracts better features by designing a network model [24]. This method used a bilinear neural network structure to extract better specific features in the image and realizes the fusion of different features extracted by the two networks to jointly realize the image classification task.

Inspired by the above methods, we propose a new non-homologous bilinear pooling network model that used two convolutional neural networks with different structures to increase the model’s feature extraction capabilities and to introduce an attention mechanism module to weaken the useless features in the image. In order to alleviate the impact of the unbalanced distribution of the data set on the predictive ability of the network model, we also added a complement cross entropy [25] loss function to the network model. Complement cross entropy can use incorrect category information to train a robust classification model with unbalanced category distribution. The experimental results show that our method has an accuracy rate of about 1 to 3 percentage points higher than other methods under the same experimental environment.

## 3. Materials and Methods

**Figure 4:**
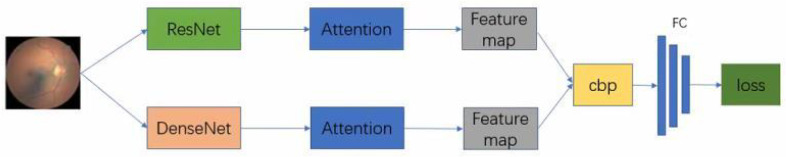
The structure of the proposed neural network model.

This paper proposes a non-homologous bilinear pooling neural network model for the classification of diabetic retinal fundus lesions. The structure of the neural network model we proposed is shown in Figure 4. It contains three parts: feature extraction module, attention mechanism module and compact bilinear pooling module. The feature extraction module is composed of ResNet and DenseNet. The image features extracted by ResNet and DenseNet is used to generate a complete data feature map after being enhanced by the attention mechanism module. The data feature maps obtained by these two neural networks are sent to the fully connected layer for classification after compact bilinear pooling. Since the network model combines two convolutional neural networks with different architectures and introduces an attention mechanism module, the feature extraction ability and prediction accuracy of the network model were greatly improved.

### 3.1. Non-Homologous Convolutional Neural Network and Compact Bilinear Pooling Operation

Due to the fine-grained nature of the diabetic retinal fundus disease data set, traditional neural networks cannot extract distinguishable image features from the data set. For the reason, this article used a bilinear pooling method [26] that performs well in fine-grained classification to classify diabetic retinopathy images. The bilinear pooling method models the high-order statistical information and regards the features in the two information streams as two different features. Performing the outer product operation and pooling operation through the features in the two information streams can capture the connection between the features in the two information streams to better obtain the global features. This method used the image translation invariant method to interactively model the local dual features and used the high-discrimination features obtained by bilinear pooling to perform image classification to obtain better results. Since different convolutional neural networks can extract different image features, we finally adopted two convolutional neural networks with different structures to extract more image features.

Although the bilinear pooling operation performs well in the field of fine-grained classification, the bilinear features are high-dimensional, usually between hundreds of thousands and millions. Such feature representations are impractical and greatly increase memory usage, making them unsuitable for subsequent analysis. Therefore, we use the compact bilinear pooling operation to perform core analysis on bilinear features to achieve dimensionality reduction. The compact bilinear pooling has the same discriminating ability as the bilinear pooling, but the dimensions are only thousands of dimensions. The compact bilinear pooling allows for back propagation of misclassified samples, enabling end-to-end optimization of image recognition. Through core analysis of the bilinear pooling, a compact bilinear representation is obtained. Compact bilinear pooling relies on the existence of low-dimensional feature maps of the kernel function. Bilinear pooling forms the global image descriptor through the following calculations:

(1) $$B\left(X\right)=\sum_{s\in S}x_{s}x_{s}^{T}$$

where *s* represents the position and $x_{s}$ is the feature vector output by CNN. The dimension of *B* is $c^{2}$. The two sets of features *X* and *Y* can be derived as follows:

(2) $$\langle B\left(X\right),B\left(Y\right)\rangle =\langle \sum_{s\in S}x_{s}x_{s}^{T},\sum_{u\in U}y_{u}y_{u}^{T}\rangle =\sum_{s\in S}\sum_{u\in U}\langle x_{s}x_{s}^{T},y_{u}y_{u}^{T}\rangle =\sum_{s\in S}\sum_{u\in U}{\langle x_{s},y_{u}\rangle }^{2}$$

where B(X) and B(Y) represent two feature vectors and $\sum_{s\in S}\sum_{u\in U}{\langle x_{s},y_{u}\rangle }^{2}$ represents a low-dimensional vector projection. If we use key(x,y) to represent the second-order polynomial kernel function. Then, we can find a low-dimensional projection function $\phi \left(x\right)\in R_{d}$ to make the value of *d* much smaller than $c^{2}$ that satisfies the following formula:

(3) 〈ϕ(x),ϕ(y)〉≈k(x,y)

where $\phi \left(x\right)\in R_{d}$ represents a projection function and key(x,y) represents a low-dimensional vector. The inner product reduced to the *d*-dimension is approximately equal to the $c^{2}$-dimensional inner product of the original. We can obtain the following representation:

(4) $$\langle B\left(X\right),B\left(Y\right)\rangle =\sum_{s\in S}\sum_{u\in U}{\langle x_{s},y_{u}\rangle }^{2}=\sum_{s\in S}\sum_{u\in U}\langle \phi \left(x\right),\phi \left(y\right)\rangle =\langle C\left(X\right),C\left(Y\right)\rangle$$

where $C\left(X\right)=\sum_{s\in S}\phi \left(X_{s}\right)$ is the compact bilinear feature, *S* is the number of channels of the vector B(X), *U* is the number of channels of the vector B(Y), *X* and *Y* are the set of local descriptors, *S* and *U* is the set of spatial positions (combination of rows and columns), $X_{s}$ and $Y_{u}$ represent local descriptors, and C(X) and C(Y) represent low-dimensional vectors after B(X) and B(Y) are mapped by the mapping function. Any polynomial kernel can create a low-dimensional approximation function to achieve dimensionality reduction.

### 3.2. Attention Mechanism Module

**Figure 5:**
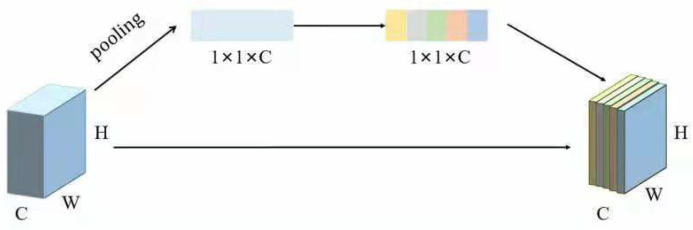
Schematic diagram of the attention module.

Although the convolutional neural network model with a non-homologous bilinear structure can extract more image features, the diabetic retinal fundus disease image contains a lot of irrelevant information, which may affect the network’s decision-making. Therefore, we used the attention mechanism to imitate the behavior of clinicians paying attention to the key features of diabetic retinal fundus lesions, thereby improving the specific feature extraction ability of the network model. The schematic diagram of the attention mechanism module is shown in Figure 5.

The attention mechanism module used the feature map output by the convolutional neural network as input. First, it performs a feature aggregation operation (global average pooling) on the input feature map to aggregate the feature map into a 1 × 1 × C feature vector. Then, the obtained feature vector is input into the fully connected layer twice after the excitation operation. The purpose of this operation is to explicitly realize the interdependence between different feature channels. This module used the attention mechanism neural network of another channel to obtain the importance of each feature channel to the feature map through self-learning and to generate a weight parameter vector. The value of the weight parameter of each channel represents the degree of attention of the neural network model to this channel. The attention mechanism can make the network model concentrate on important image features and weakens the impact of useless features. Therefore, after adopting the attention mechanism module, the feature distinctiveness of the bilinear network model is better, which can further improve the predictive ability of the network model. The global average pooling is defined as follows:

(5) $$v=F_{GAP}\left(x\right)=\frac{1}{H\times W}\sum_{i=1}^{W}\sum_{j}^{H}x\left(i,j\right)$$

where *H* and *W* are the height and width of the feature map, x(i,j) represents each pixel of the feature map and *v* is the output feature vector of the attention mechanism. The obtained feature vector is processed by two fully connected layers and activation function. The specific reference formula is defined as follows:

(6) $$y=F\left(v,W\right)=\delta \left(W_{2}\sigma \left(W_{1}v\right)\right)$$

where $W_{1}$ and $W_{2}$ represent the weight of the fully connected layer, v is the feature vector obtained after global average pooling, δ represents the ReLU activation function and σ represents the Sigmoid activation function. The obtained feature vector y is multiplied by the input feature map x channel by channel to obtain the attention feature map, which is the final output feature map.

### 3.3. The Problem of Unbalanced Sample Distribution

In clinical medicine, the proportion of patients with serious illnesses accounts for a very small part of the total number of people examined, which causes the acquired data set to have a serious uneven distribution problem. When using the unbalanced data for network learning, the class with a large number of samples dominates the training process [27,28,29]. The learned classification models tend to perform better in classes with a large number of samples but perform poorly in classes with a small number of samples. Complement cross entropy can be used to solve the problem of network performance degradation caused by unbalanced data sets. The complement cross entropy is driven by the training of non-label category samples. It does not need to increase the number of samples or to increase the size of the minority losses. On the contrary, this method used the information of the incorrect class to train a robust classification model of the unbalanced class distribution. Therefore, this strategy can provide better learning opportunities, especially for categories with a small sample size, because it encourages the correct category (including a few categories) to be greater than the SoftMax score in all other incorrect categories. The complement entropy loss function *C* is calculated from the average value of the sample entropy of the incorrect class in each batch. Complement cross entropy is defined as follows:

(7) $$C\left(y,\hat{y}\right)=-\frac{1}{N}\sum_{i=1}^{N}\sum_{j=1,j\neq g}^{K}\frac{{\hat{y}}^{\left(i)[j\right]}}{1-{\hat{y}}^{\left(i)[g\right]}}\log\frac{{\hat{y}}^{\left(i)[j\right]}}{1-{\hat{y}}^{\left(i)[g\right]}}$$

where *N* is the number of samples in the mini-batch, *K* is the total number of categories, $\hat{y}$ is the estimated probability vector of the category of a given input sample, *g* is the sequence number of the groudtruth category and ${\hat{y}}^{\left(i)[j\right]}$ is the prediction that the *i*th given input sample is the *g* category probability. The purpose of this entropy is to predict the probability of the ground truth class being greater in other incorrect classes. The way to achieve this goal is to split the SoftMax scores across the wrong category. This means that the more neutral the prediction probability distribution of the incorrect class, the more confident the prediction of the correct class. Therefore, the optimizer should maximize the complement entropy because the entropy will be maximized when the probability distribution is uniform. The balanced complement entropy loss function is used to match the scale between cross entropy and complementary entropy. It is defined as follows:

(8) $$\bar{C}\left(y,\hat{y}\right)=\frac{1}{K-1}C\left(y,\hat{y}\right)$$

where $\frac{1}{K-1}$ is the balance coefficient. In order to balance cross entropy and complement entropy, an adjustment factor γ is added. The final loss function is expressed as follows:

(9) $$L=H\left(y,\hat{y}\right)+\frac{\gamma }{K-1}C\left(y,\hat{y}\right)$$

The advantage of the loss over focal loss is that it does not require setting weights to balance the internal relationship of loss. The loss function can solve the problem of sample imbalance through the relationship of the sample itself.

## 4. Experiments and Results

### 4.1. Data Set

**Figure 6:**
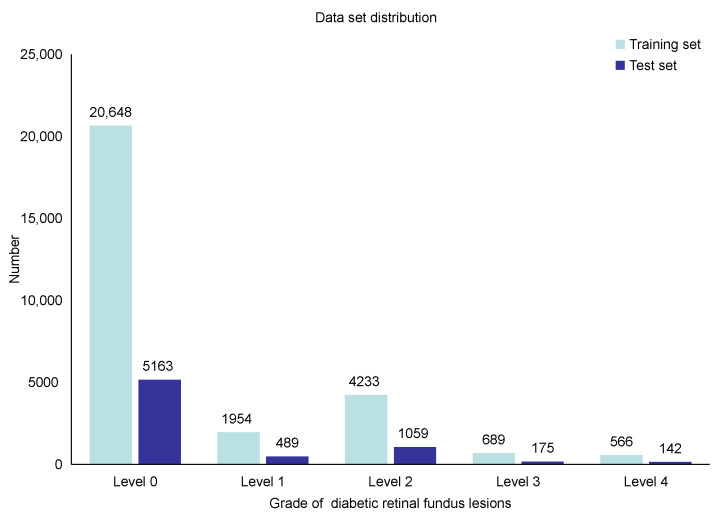
The distribution of images of each category in the training set and test sets.

In the experiment, we used Kaggle’s Diabetic Retinopathy Detection Challenge (EyePACS) data set to evaluate the performance of the network model proposed in this paper. The EyePACS data set has a total of 35K images, which were taken under different conditions and equipment. The distribution of the data set is shown in Figure 6. From the figure, we can see that there is a serious imbalance in the distribution of the data set.

### 4.2. Ablation Test

**Figure 7:**
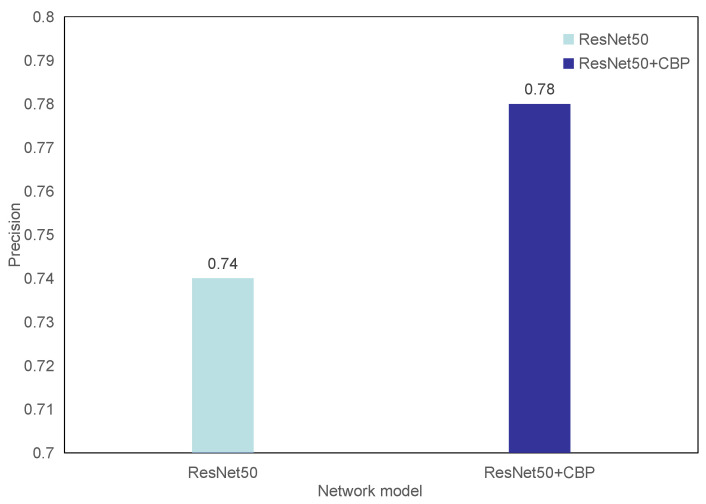
Comparison of compact bilinear pooling model.

The bilinear pooling method is a very effective method in the field of fine-grained classification [7,11,12]. This paper treats the image classification problem of diabetic retinal fundus lesions from the perspective of fine-grained image classification and introduces a bilinear pooling model. We tested the performance improvement of the CNN model after compact bilinear pooling on the diabetic retinal fundus image data set. In the experiment, we used the same data stream instead of one data stream except that the two methods used exactly the same experimental steps. The experimental results are shown in Figure 7. From the figure, we can see that bilinear pooling improves the prediction accuracy of the network model from 0.74 to 0.78. From the experimental results, it can be concluded that bilinear pooling, a very effective method in the field of fine-grained classification, is also suitable for the classification of diabetic retinal fundus lesions in the medical field.

**Figure 8:**
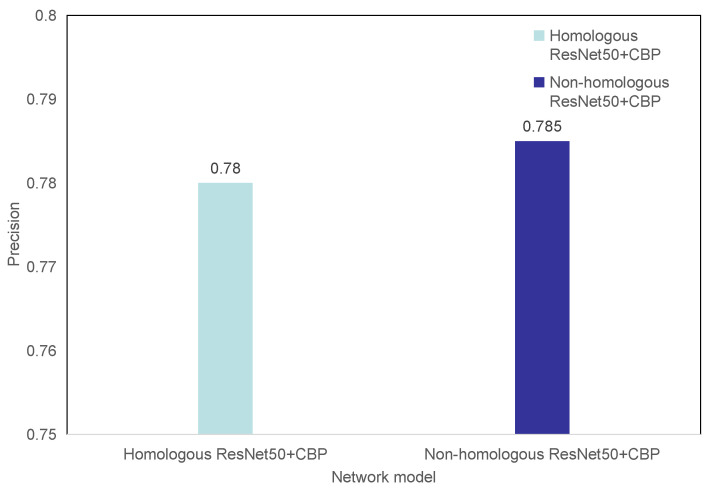
Comparison of homologous bilinear pooling and non-homologous bilinear pooling.

We also performed experiments on the homologous bilinear pooling and non-homologous bilinear pooling methods on the data set. In addition, these two methods use exactly the same experimental steps. The experimental results are shown in Figure 8. Compared with the homologous bilinear pooling, the non-homologous bilinear pooling improves the prediction accuracy of the network model from 0.780 to 0.785. From the experimental results, it can be seen that, in the grading experiment of diabetic retinopathy, the non-homologous double linear pooling is a better method. Non-homologous bilinear pooling trains two convolutional neural networks with different parameter weights at the same time. Different data streams are used to recognize different image features, which is more helpful to extract more specific diabetic retinopathy features.

**Figure 9:**
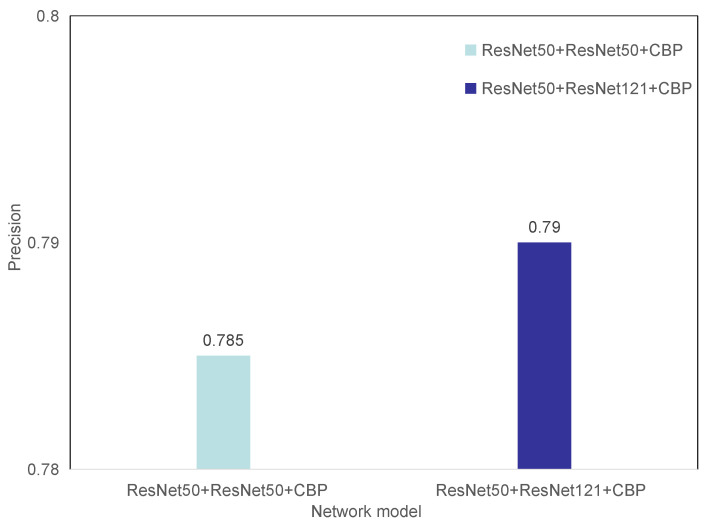
Comparison of different convolutional neural network structures.

We tried to turn one of the two ResNet50 networks in the non-homologous bilinear pooling model into a DenseNet network and conducted a comparative test under the same experimental conditions. The experimental results are shown in Figure 9. It can be seen from the experimental results that different convolutional neural network models combined with bilinear pooling perform better in grading diabetic retinal fundus images. Different convolutional neural network models can extract more distinctive features in the data set images.

**Figure 10:**
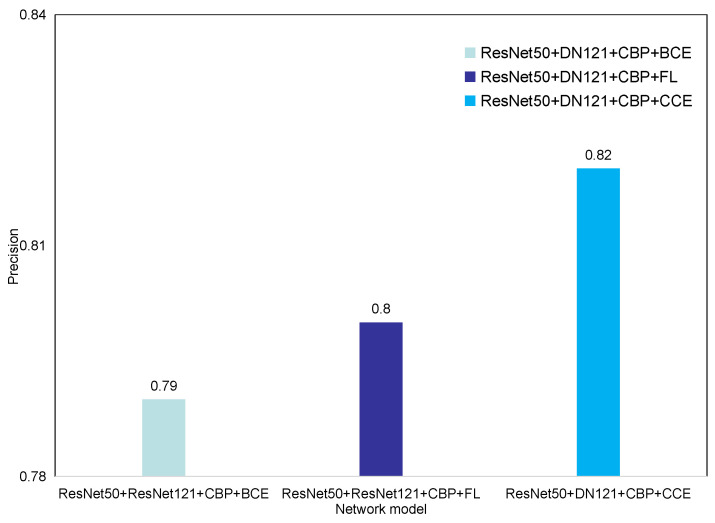
Comparison of different loss functions.

We compare cross-entropy loss and local loss with CCE loss [29]. Except for the difference in the loss function, these three experimental methods use exactly the same experimental steps. The experimental results are shown in Figure 10. From the figure, we can see that focal loss increases the accuracy from 0.79 to 0.80 and that CCE loss increases the accuracy to 0.82. The experimental data shows that CCE loss outperforms focal loss in solving the problem of uneven distribution of data sets.

**Figure 11:**
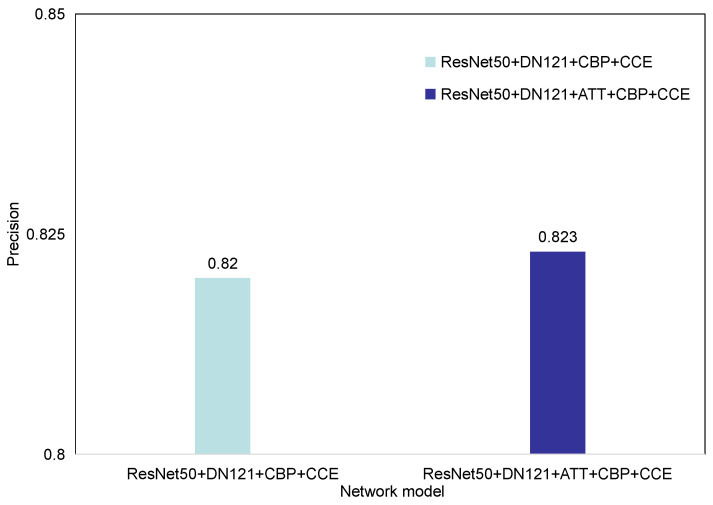
Comparison of attention mechanism modules.

Figure 11 lists the effects of adding attention mechanism after the convolutional neural network model on the experiment. We conducted a bilinear pooling experiment and a bilinear pooling method experiment with the attention mechanism on the EyePACS data set. In addition, these two methods use exactly the same experimental procedures. It can be seen from the table that the bilinear pooling model after adding the attention mechanism improves the prediction accuracy of the network from 0.820 to 0.823. It can be seen from the above data that, in the grading experiment of diabetic retinal fundus disease, the attention mechanism has a more obvious influence on the image classification performance. As mentioned above, at the model level, the attention mechanism can classify the features extracted by CNN channel by channel and finds out the dependencies between each channel. At the sample level, the setting of two attention mechanisms can make the network pay different attention to different image features. The image features enhance the expressive ability of the network model, thereby improving the predictive ability of the network.

**Figure 12:**
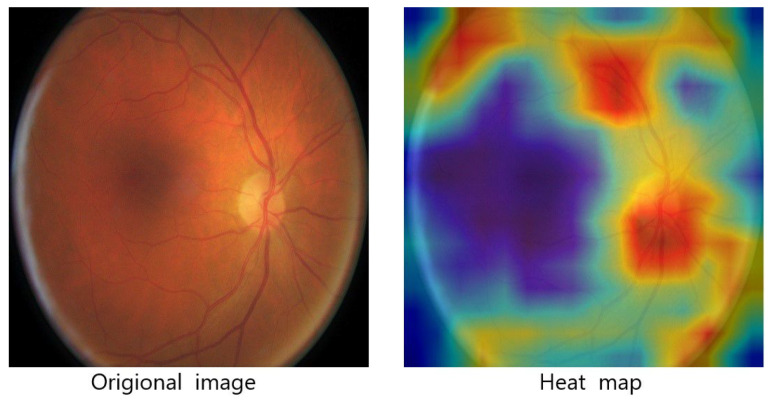
The visualization of attention mechanisms.

Figure 12 visually visualizes the degree of attention our network model pays to different regions in the input image after using the attention mechanism. The image on the left is an original image in the data set used in the experiment and the image on the right is a visual display of the network model’s attention to different areas of the input image. In the heat map, red means that the image feature of the region has a higher weight and blue means that the feature of the corresponding region has a lower weight.

### 4.3. Evaluation Index and the Experimental Evaluation Result of Our Network

In order to evaluate the classification performance of the model, we use accuracy as an experimental evaluation index. The accuracy formula is defined as follows:

(10) $$\;\mathrm{Accuracy}\;=\frac{1}{\left|D\right|}\sum_{i=1}^{\left|D\right|}\frac{\left|Y_{i}\cap X_{i}\right|}{\left|Y_{i}\cup X_{i}\right|}$$

where $Y_{i}$ represents the label of the first sample and $X_{i}$ is the prediction result of the *i*th sample.The network model of the present invention is evaluated by commonly used machine learning evaluation indicators. The results obtained are shown in Table 1:

### 4.4. The Experimental Environment and the Experimental Results

In order to evaluate the enhancement effect of our proposed method, we tested our proposed method several times on the EyePACS data set and compared it with two state-of-the-art methods: BIRA-Net [30] and SEA-Net [31]. BIRA-Net is also an algorithm improvement based on bilinear pooling. It extracts advanced features through repeated combinations of CNN, attention module and bilinear pooling module. SEA-Net used the combination of ResNet and attention mechanism to improve the model’s ability to extract features. In addition, we also compared the two network models with ResNet50 and DenseNet121. In order to ensure the accuracy of each method, we downloaded the source code of BIRA-Net, SEA-Net, ResNet50 and DenseNet121 and tested all network models with the same experimental environment and data set. The hardware and software environment used in the experiment is shown in Table 2.

The final test results of several methods are shown in Table 3. It can be seen from the table that the method proposed in this paper is 0.8% higher than BIRA-Net and 0.4% higher than SEA-Net. Compared with the baseline, the method proposed in this paper greatly improves the experimental performance, which is 8.3% higher than ResNet50 and 2.6% higher than DenseNet121. The comparison results show that our proposed network architecture is more suitable for the field of diabetic retinopathy fundus image classification.

## 5. Conclusions

In this paper, we solved the classification problem of diabetic retinal fundus lesions from the perspective of fine-grained classification and proposed a novel non-homologous bilinear pooling network model. The final prediction accuracy of the network model we proposed reached the most advanced level. In this network model, we used two convolutional neural networks with different structures to increase the image feature extraction capability of the network model. At the same time, we also used the attention mechanism to enable the network model to focus on useful information and to weaken the influence of useless feature information on the predictive ability of the network model. The image features extracted by the bilinear structure network are high-dimensional, which affects the calculation overhead of the subsequent feature analysis and the space occupancy rate of the memory. Therefore, we used a compact bilinear pool operation to perform core analysis on bilinear features to achieve dimensionality reduction. The compact bilinear pooling operation allows our network model to greatly reduce the memory usage while maintaining the prediction accuracy.

Collectively, the bilinear network model structure and attention mechanism are both used to increase the feature extraction ability of the network model. The key factor to improve the accuracy of network prediction is the image features extracted by the network. Only when the network model can fully extract those distinguishing characteristics and can make judgments based on these characteristics can the network model be considered able to distinguish things. In the field of image classification and image recognition, future research should continue to focus on how to further improve the specific feature extraction capabilities of network models.

## Figures and Tables

**Table 1:** The results predicted by our network model.

Evaluation Index	Accuracy	Recall Rate	Precision	(F1-Score)
Result	0.823	0.819	0.828	0.823

**Table 2:** The hardware and software environment used in the experiment.

Hardware or Software Environment	Name
Sytstem Version	Ubuntu 16.04
CPU	Intel(R) Xeon(R) W-2150B CPU @ 3.00 GHz
GPU	NVIDIA RTX2080TI
Framework	Pytorch 1.4.0
Driver	440.82
Cuda	10.2

**Table 3:** The comparison of experimental results of different network models.

Method	Accuracy	Precision	Recall Rate	(F1-Score)
ResNet50	0.740	0.723	0.791	0.755
DenseNet121	0.797	0.714	0.793	0.751
BIRA-Net	0.815	0.749	0.816	0.781
SEA-Net	0.819	0.787	0.824	0.805
Ours	0.823	0.819	0.828	0.823

## References

[1] Araújo T., Aresta G., Mendonça L., Penas S., Maia C., Carneiro Â., Mendonça A.M., Campilho A. (2020). DR|GRADUATE: Uncertainty-aware deep learning-based diabetic retinopathy grading in eye fundus images—ScienceDirect. Med. Image Anal..

[2] Peng J., Wang Y. (2021). Medical Image Segmentation with Limited Supervision: A Review of Deep Network Models. IEEE Access.

[3] Wah C., Branson S., Welinder P., Perona P., Belongie S. (2011). The Caltech-UCSD Birds-200-2011 Dataset. http://www.vision.caltech.edu/visipedia/CUB-200-2011.html.

[4] Lin T.Y., Goyal P., Girshick R., He K., Dollár P. Focal Loss for Dense Object Detection. Proceedings of the 2017 IEEE International Conference on Computer Vision (ICCV).

[5] Nguyen H., Butler M., Roychoudhry A., Shannon A., Flack J., Mitchell P. Classification of diabetic retinopathy using neural networks. Proceedings of the 18th Annual International Conference of the IEEE Engineering in Medicine and Biology Society.

[6] Xiaohui Z., Chutatape A. Detection and classification of bright lesions in color fundus images. Proceedings of the 2004 International Conference on Image Processing (ICIP ’04).

[7] Zhang X., Chutatape O. A SVM approach for detection of hemorrhages in background diabetic retinopathy. Proceedings of the 2005 IEEE International Joint Conference on Neural Networks.

[8] Zhang X., Chutatape O. Top-down and bottom-up strategies in lesion detection of background diabetic retinopathy. Proceedings of the 2005 IEEE Computer Society Conference on Computer Vision and Pattern Recognition (CVPR ’05).

[9] Barriga E.S., Murray V., Agurto C., Pattichis M.S., Bauman W., Zamora G., Soliz P. Automatic system for diabetic retinopathy screening based on AM-FM, partial least squares, and support vector machines. Proceedings of the 2010 IEEE International Symposium on Biomedical Imaging: From Nano to Macro.

[10] Xu L., Luo S. Support vector machine based method for identifying hard exudates in retinal images. Proceedings of the IEEE Youth Conference on Information, Computing & Telecommunication.

[11] Sinthanayothin C., Kongbunkiat V., Phoojaruenchanachai S., Singalavanija A. Automated screening system for diabetic retinopathy. Proceedings of the 3rd International Symposium on Image and Signal Processing and Analysis (ISPA 2003).

[12] Acharya R., Chua C.K., Ng E.Y., Yu W., Chee C. (2008). Application of higher order spectra for the identification of diabetes retinopathy stages. J. Med. Syst..

[13] Pratt H., Coenen F., Broadbent D.M., Harding S.P., Zheng Y. (2016). Convolutional Neural Networks for Diabetic Retinopathy. Procedia Comput. Sci..

[14] Kori A., Chennamsetty S.S., Alex V. (2018). Ensemble of convolutional neural networks for automatic grading of diabetic retinopathy and macular edema. arXiv.

[15] Olivas E., Guerrero J., Martinez-Sober M., Magdalena-Benedito J., Serrano L¢pez A. (2010). Handbook of Research on Machine Learning Applications and Trends: Algorithms, Methods, and Techniques.

[16] Wang Z., Yang J. Diabetic Retinopathy Detection via Deep Convolutional Networks for Discriminative Localization and Visual Explanation. Proceedings of the Workshops at the Thirty-Second AAAI Conference on Artificial Intelligence.

[17] Bravo M.A., Arbeláez P.A. Automatic diabetic retinopathy classification. Proceedings of the 13th International Symposium on Medical Information Processing and Analysis.

[18] Fu J., Zheng H., Mei T. Look closer to see better: Recurrent attention convolutional neural network for fine-grained image recognition. Proceedings of the IEEE Conference on Computer Vision and Pattern Recognition.

[19] Hu J., Shen L., Sun G. (2017). Squeeze-and-Excitation Networks. arXiv.

[20] Huang G., Liu Z., Laurens V.D.M., Weinberger K.Q. (2016). Densely Connected Convolutional Networks. arXiv.

[21] Simonyan K., Zisserman A.J. (2014). Very Deep Convolutional Networks for Large-Scale Image Recognition. arXiv.

[22] Szegedy C., Liu W., Jia Y., Sermanet P., Rabinovich A. (2014). Going Deeper with Convolutions. arXiv.

[23] Targ S., Almeida D., Lyman K. (2016). Resnet in Resnet: Generalizing Residual Architectures. arXiv.

[24] Lin T.Y., Roychowdhury A., Maji S. (2017). Bilinear Convolutional Neural Networks for Fine-grained Visual Recognition. IEEE Trans. Pattern Anal. Mach. Intell..

[25] Kim Y., Lee Y., Jeon M. (2020). Imbalanced Image Classification with Complement Cross Entropy. arXiv.

[26] Lin T.Y., Roychowdhury A., Maji S. (2015). Bilinear CNN Models for Fine-grained Visual Recognition. arXiv.

[27] Kang B., Xie S., Rohrbach M., Yan Z., Gordo A., Feng J., Kalantidis Y. (2019). Decoupling representation and classifier for long-tailed recognition. arXiv.

[28] Tang K., Huang J., Zhang H. (2020). Long-tailed classification by keeping the good and removing the bad momentum causal effect. arXiv.

[29] Zhou B., Cui Q., Wei X.S., Chen Z.M. BBN: Bilateral-branch network with cumulative learning for long-tailed visual recognition. Proceedings of the IEEE/CVF Conference on Computer Vision and Pattern Recognition.

[30] Zhao Z., Xu X. BiRA-Net: Bilinear Attention Net for Diabetic Retinopathy Grading. Proceedings of the 2019 IEEE International Conference on Image Processing (ICIP).

[31] Zhao Z., Chopra K., Zeng Z., Li X. Sea-Net: Squeeze-And-Excitation Attention Net for Diabetic Retinopathy Grading. Proceedings of the 2020 IEEE International Conference on Image Processing (ICIP).

